# Association between alcohol consumption and incidence of type 2 diabetes in middle-aged Japanese from Panasonic cohort study 12

**DOI:** 10.1038/s41598-024-71383-6

**Published:** 2024-09-02

**Authors:** Fuyuko Takahashi, Hiroshi Okada, Yoshitaka Hashimoto, Kazushiro Kurogi, Hiroaki Murata, Masato Ito, Michiaki Fukui

**Affiliations:** 1 Department of Endocrinology and Metabolism, Graduate School of Medical Science, Kyoto Prefectural University of Medicine, 465 Kajii-Cho, Kawaramachi-Hirokoji, Kamigyo-Ku, Kyoto, 602-8566 Japan; 2 Department of Diabetes and Endocrinology, Matsushita Memorial Hospital, 5-55 Sotojima-Cho, Moriguchi, 570-8540 Japan; 3 Department of Health Care Center, Panasonic Health Insurance Organization, 5-55 Sotojima-Cho, Moriguchi, 570-8540 Japan; 4 Department of Orthopaedic Surgery, Matsushita Memorial Hospital, 5-55 Sotojima-Cho, Moriguchi, 570-8540 Japan

**Keywords:** Type 2 diabetes, Obesity, Alcohol consumption, Metabolic disorders, Risk factors, Endocrine system and metabolic diseases

## Abstract

This retrospective cohort study aimed to investigate the association between alcohol consumption and the onset of type 2 diabetes in middle-aged Japanese individuals. Participants were aged 40 and above from Panasonic Corporation, Osaka, Japan’s medical health checkup program from 2008 to 2021. Alcohol consumption was calculated by converting the quantity consumed into daily ethanol consumption. We assessed the association between alcohol consumption and the onset of type 2 diabetes using Cox regression analysis. The total and median follow-up duration was 13 years and 7 (3–13) years (748,090 person-years). Among 102,802 participants, 7,510 participants (7.3%) developed type 2 diabetes during the study period. Alcohol consumption at the level of 0 < to < 22 g/day and 22 to < 39 g/day were negatively associated with developing type 2 diabetes compared to complete alcohol abstainers. Alcohol consumption at levels of 39 to < 66 g/day and at levels of ≥ 66 g/day were positively associated with developing type 2 diabetes in participants with BMI < 25 kg/m^2^. All levels of alcohol consumption were negatively associated with developing type 2 diabetes in participants with BMI ≥ 25 kg/m^2^. Moderate-to-heavy alcohol consumption were positively associated with developing type 2 diabetes for participants with BMI < 25 kg/m^2^, whereas alcohol intake was negatively associated with developing type 2 diabetes among participants with BMI ≥ 25 kg/m^2^.

## Introduction

Globally, the prevalence of diabetes is rapidly increasing. The prevalence of diabetes in 20–79 years in 2021 was estimated to be 536.6 million people in the world, rising to 783.2 million in 2045^1^. Japan is the country with the ninth largest population with diabetes in the world. Individuals with type 2 diabetes have a high risk of complications, including cardiovascular diseases and sarcopenia^2,3^. Therefore, it is important to prevent the development of type 2 diabetes. Improvement in dietary habits, including alcohol consumption and exercise, is crucial for preventing the onset of diabetes^4–6^.

Several meta-analyses have focused on Western populations regarding the association between alcohol consumption and the development of diabetes^7–13^. Alcohol consumption improves insulin sensitivity and reduces HbA1c levels^7^. Daily alcohol consumption < 24 g was negatively associated with developing type 2 diabetes compared with non-drinkers, whereas daily alcohol consumption > 24 g was not related to developing type 2 diabetes^8^. Daily alcohol consumption of 12–24 g/day was negatively related to developing type 2 diabetes^13^. The relative risk of type 2 diabetes was lowest, with a daily alcohol intake of 22 g in males and 24 g in females^9^. Daily alcohol consumption of less than 48 g^10^ or 63 g^11^ was negatively associated with developing type 2 diabetes. The negative association was observed to be less than 49 g in females^12^. In summary, light or moderate alcohol consumption may be negatively associated with the onset of type 2 diabetes in individuals from Western societies. However, previous studies focusing on Asian populations have reported varying results. One meta-analysis suggested that daily alcohol consumption > 57 g/day was positively associated with developing type 2 diabetes in Asian men^14^. Observational studies have reported the relationship between alcohol consumption and the onset of type 2 diabetes in Asian populations. Some reports have suggested that alcohol consumption is positively associated with developing type 2 diabetes^15–19^. Some studies have suggested that alcohol consumption is negatively associated with developing type 2 diabetes^20–22^. A report also suggested that alcohol consumption is not associated with the development of type 2 diabetes^23^. Other reports have suggested a U-shaped association between alcohol consumption and the onset of type 2 diabetes^24,25^. In summary, the relationship between alcohol consumption and onset of diabetes in Asians remains controversial. The discrepancies in these reports among Asians can be attributed to a lack of consideration of blood tests, small sample sizes, and short observation periods in individual studies. Moreover, the Asian population has a lower body mass index (BMI) and insulin resistance than the Western population. Additionally, the BMI of patients with diabetes is not remarkably greater than that of the general population in the Asian population, who tend to have reduced insulin secretion rather than insulin resistance^26^, suggesting that the relationship between alcohol consumption and the onset of diabetes might differ between these two populations. Therefore, in this study, we evaluated the association between alcohol consumption and the onset of type 2 diabetes in a large-scale, long-term cohort of Japanese individuals.

## Results

**Fig. 1:**
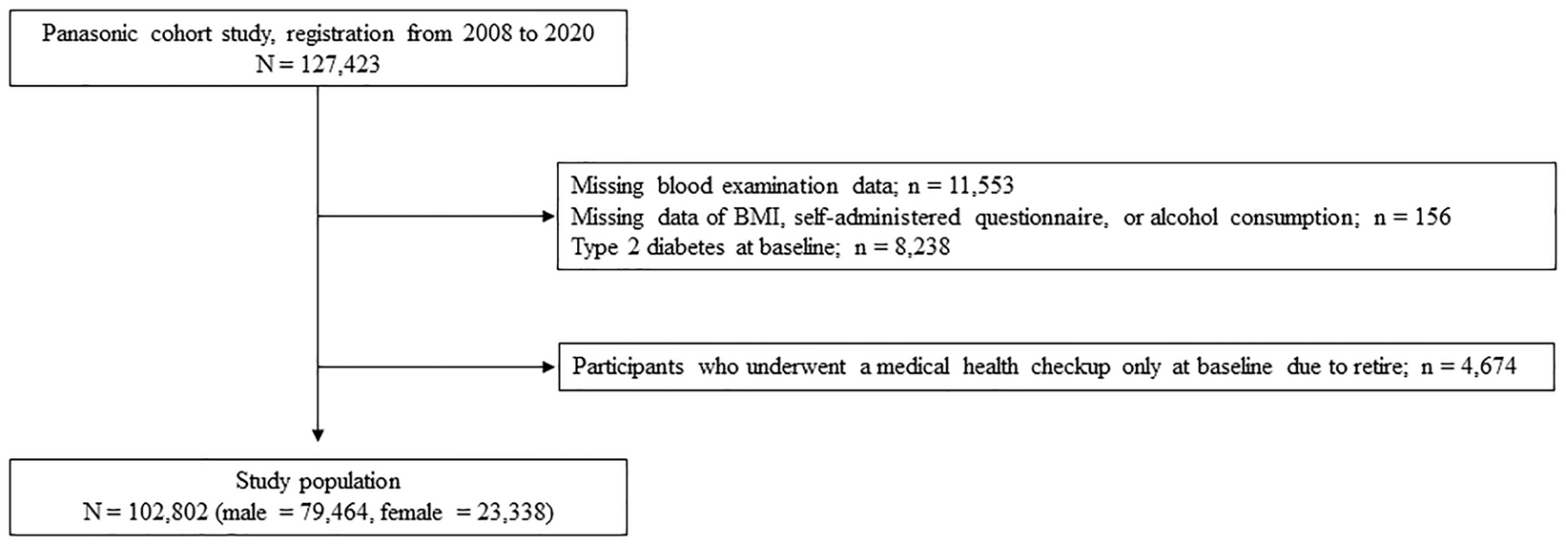
Flow diagram of participants’ registration.

**Fig. 2:**
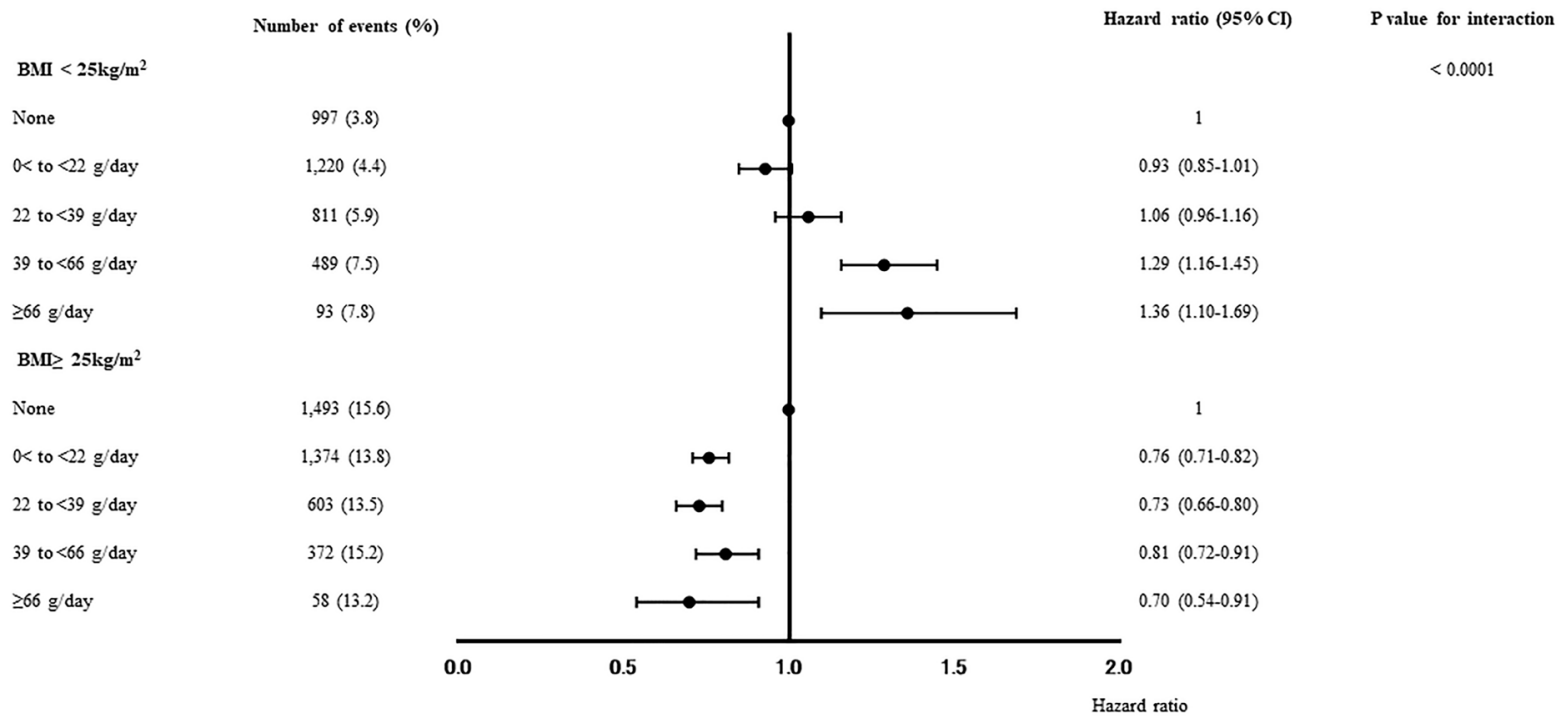
Adjusted hazard ratios of alcohol consumption for incidence of type 2 diabetes according to BMI category adjusted for age, sex, smoking status, and exercise habits.

In total, 102,802 participants were enrolled (Fig. 1). Table 1 displays the baseline characteristics of the research participants. The average age and BMI were 47.9 ± 5.8 years and 23.2 ± 3.3 kg/m^2^, respectively. The proportions of daily alcohol consumption were 35.1% for 0 g/day, 36.9% for 0 < to < 22 g/day, 17.7% for 22 to < 39 g/day, 8.7% for 39 to < 66 g/day, and 1.6% for ≥ 66 g/day. Table 2 displays type 2 diabetes incident cases and incidence rates by alcohol usage. The median follow-up duration was 7 (3–13) years (748,090 person-years). During the study period, 7,510 participants developed type 2 diabetes. The incidence rates of type 2 diabetes were 6.9% for daily alcohol consumption of 0 g/day, 6.8% for 0 < to < 22 g/day, 7.8% for 22 to < 39 g/day, 9.6% for 39 to < 66 g/day, and 9.2% for ≥ 66 g/day in whole participants. The adjusted hazard ratios (HRs) for incident type 2 diabetes are shown in Table 3. Multivariable analysis showed the HR of alcohol consumption at the level of 0 < to < 22 g/day (HR, 0.85; 95% CI, 0.81–0.90) and 22 to < 39 g/day (HR, 0.92; 95% CI, 0.86–0.99) were lower than those of 0 g/day. The HR of alcohol consumption at the level of 39 to < 66 g/day and ≥ 66 g/day were 0.99 (95% CI, 0.83–1.18) and 1.06 (95% CI, 0.90–1.25), respectively. The results of the subgroup analysis, examining the effects of BMI, FPG, and sex on the association between alcohol consumption levels and incidence of type 2 diabetes, are shown in Figs. 2, Supplementary Figs 1 and 2. There was an interaction effect (P for interaction < 0.0001) between alcohol consumption and BMI. In participants with BMI < 25 kg/m^2^, the HR of alcohol consumption levels of 39 to < 66 g/day and ≥ 66 g/day were higher than those of 0 g/day. On the other hand, the HR of alcohol consumption levels of 0 to < 22 g/day, 22 to < 39 g/day, 39 to < 66 g/day, and ≥ 66 g/day were lower than those of 0 g/day in individuals with BMI ≥ 25 kg/m^2^. We found no interaction effect between alcohol consumption and FPG category (P for interaction = 0.13) and sex (P for interaction = 0.42).

**Table 1:** Characteristics of participants at baseline.

N	102,802
Age (y)	47.9 (5.8)
Male, n, (%)	79,464 (77.3)
Body mass index (kg/m^2^)	23.2 (3.3)
Systolic blood pressure (mmHg)	120.0 (15.0)
Diastolic blood pressure (mmHg)	75.5 (11.1)
Aspartate aminotransferase (IU/L)	22.5 (12.0)
Alanine aminotransferase (IU/L)	24.5 (18.2)
Low-density lipoprotein cholesterol (mg/dL)	126.5 (31.1)
High-density lipoprotein cholesterol (mg/dL)	60.5 (15.6)
Triglycerides (mg/dL)	115.8 (90.7)
Fasting plasma glucose (mg/dL)	93.9 (9.4)
Uric acid (mg/dL)	5.8 (1.4)
Smoking (none/past/current), n, (%)	52,324/15,853/34,625 (50.9/15.4/33.7)
Alcohol consumption (none /0 < to < 22 /22 to < 39 /39 to < 66 / ≥ 66 g/day), n, (%)	36,092/37,911/18,203/8,960/1,636 (35.1/36.9/17.7/8.7/1.6)
Physical exercise, n, (%)	18,361 (17.9)

Data are presented as mean (standard deviation, or percentage) or absolute number.

**Table 2:** Incident cases and incident rate for type 2 diabetes according to alcohol consumption.

	0 g/day	0 < to < 22 g/day	22 to < 39 g/day	39 to < 66 g/day	≥ 66 g/day	Total
Whole, n	36,092	37,911	18,203	8960	1636	10,2802
Incident diabetes (%)	2,490 (6.9)	2,594 (6.8)	1,414 (7.8)	861 (9.6)	151 (9.2)	7,510 (7.3)
BMI < 25 kg/m^2^, n	26,521	27,984	13,736	6509	1197	75,947
Incident diabetes (%)	997 (3.8)	1220 (4.4)	811 (5.9)	489 (7.5)	93 (7.8)	3,610 (4.8)
BMI** ≥ **25 kg/m^2^, n	9571	9927	4467	2451	439	26,855
Incident diabetes (%)	1,493 (15.6)	1,374 (13.8)	603 (13.5)	372 (15.2)	58 (13.2)	3,900 (14.5)
FPG < 100 mg/dL, n	29,034	29,001	12,548	5944	1123	77,650
Incident diabetes (%)	823 (2.8)	744 (2.6)	314 (2.5)	200 (3.4)	43 (3.8)	2,124 (2.7)
FPG ≥ 100 mg/dL, n	7058	8910	5655	3016	513	25,152
Incident diabetes (%)	1667 (23.6)	1850 (20.8)	1,100 (19.5)	661 (21.9)	108 (21.1)	5,386 (21.4)
Male, n	22,517	30,441	16,544	8432	1,530	79,464
Incident diabetes (%)	2,053 (9.1)	2431 (8.0)	1379 (8.3)	851 (10.1)	147 (9.6)	6,861 (8.6)
Female, n	13,575	7470	1659	528	106	23,338
Incident diabetes (%)	437 (3.3)	163 (2.2)	35 (2.1)	10 (1.9)	4 (3.8)	649 (2.8)

*BMI* body mass index, *FPG* fasting plasma glucose.

**Table 3:** Adjusted hazard ratios for incidence of type 2 diabetes during follow-up period in whole participants.

	HR (95% CI)
Age (per 1 years)	1.07 (1.07–1.08)
Sex (ref: female)	1.64 (1.51–1.79)
Body mass index (per 1 kg/m^2^)	1.20 (1.19–1.20)
Smoking (past) (ref: none)	1.09 (1.02–1.17)
Smoking (current) (ref: none)	1.44 (1.37–1.51)
Alcohol consumption (ref: 0 g/day)	1
0 < to < 22 g/day	0.85 (0.81–0.90)
22 to < 39 g/day	0.92 (0.86–0.99)
39 to < 66 g/day	0.99 (0.83–1.18)
≥ 66 g/day	1.06 (0.90–1.25)
Physical exercise (yes) (ref: no)	0.97 (0.91–1.03)

Adjusted for age, sex, body mass index, smoking status, and exercise habits.

Supplementary Fig. 3 showed the sensitivity analysis examining the effects of BMI on the association between alcohol consumption levels and the incidence of type 2 diabetes after excluding incident cases that occurred within the first two years of the follow-up. The results were nearly equivalent to the results of the overall analysis. Supplementary Fig. 4 showed the sensitivity analysis examining the effects of BMI on the association between alcohol consumption levels and the incidence of type 2 diabetes after adding BMI as a continuous variable covariate. The results were nearly equivalent to the findings of the analysis without adding BMI as a continuous variable covariate. Supplementary Fig. 5 shows the sensitivity analysis according to BMI category of BMI < 23 kg/m^2^ and ≥ 23 kg/m^2^. The results were nearly equivalent to those of the BMI category of BMI < 25 kg/m^2^ and ≥ 25 kg/m^2^.

## Discussion

The major findings of this study are that alcohol consumption at the level of 0 to < 22 g/day and 22 to < 39 g/day were negatively associated with developing type 2 diabetes in middle-aged Japanese. In participants with BMI < 25 kg/m^2^, alcohol consumption levels of 39 to < 66 g/day and ≥ 66 g/day were positively associated with developing type 2 diabetes. In the participants with BMI ≥ 25 kg/m^2^, alcohol consumption were negatively associated with developing type 2 diabetes. No interaction effects were found between alcohol consumption, and FPG category and sex on type 2 diabetes onset.

Although the mechanism through which alcohol consumption affects developing diabetes remains unknown, some studies have reported an association between alcohol consumption and insulin resistance. Alcohol consumption can improve insulin resistance^27–30^. Alcohol consumption increases insulin sensitivity by increasing adiponectin and leptin levels^31,32^. Previous studies showed that high levels of adiponectin are closely associated with increased insulin sensitivity^33–35^. A previous study demonstrated that low fasting plasma adiponectin levels are linked with reduced insulin-stimulated skeletal muscle insulin receptor tyrosine phosphorylation, which could be a potential cause of decreased insulin sensitivity^36^. Alcohol also has anti-inflammatory properties. A previous study found that alcohol consumption had a U-shaped association with C-reactive protein levels^37^. Furthermore, moderate ethanol consumption inhibits interleukin-6 production or activity^38^. Inflammation is also associated with insulin resistance^39^. Moreover, alcohol consumption may indirectly affect adipocytes because it has long been known that acetate, the chief circulating metabolite of alcohol, has an antilipolytic effect on adipocytes^38,40^. The ability of alcohol consumption to acutely lower free fat acid levels presumably reflects the generation of hepatic acetate^41^. Therefore, alcohol consumption may prevent insulin resistance by suppressing adipocyte lipolysis, resulting in reduced levels of circulating free fatty acids. On the other hand, alcohol consumption was associated with a significant decrease in the insulin secretion level at the alcohol consumption level of ≥ 40 g/day^42^. Excessive alcohol consumption can lead to pancreatic fibrosis^43^ and, depending on the amount, may potentially result in decreased insulin secretion due to alcohol intake.

In this study, different results were obtained after stratification according to BMI. In participants with BMI < 25 kg/m^2^, moderate-to-high alcohol consumption was positively associated with developing type 2 diabetes. In participants with BMI ≥ 25 kg/m^2^, alcohol consumption was negatively associated with developing type 2 diabetes. As mentioned earlier, alcohol consumption is associated with improving insulin resistance. Therefore, it might exert a negative association with the onset of diabetes in individuals with BMI ≥ 25 kg/m^2^, who are anticipated to have higher insulin resistance. Conversely, individuals with BMI < 25 kg/m^2^ who are not anticipated to have high insulin resistance may be less likely to benefit from this effect and may even face positive association with developing diabetes due to decreased insulin secretion caused by alcohol consumption. Previous reports have suggested that the impact of alcohol on type 2 diabetes may vary depending on the BMI in Japanese individuals^44–47^. Waki. et al.^45^ reported that moderate to high alcohol consumption was positively associated with developing type 2 diabetes in the participants with BMI ≤ 22 kg/m^2^, but not in the participants with BMI ≥ 22 kg/m^2^. Tsumura et al.^46^ reported that moderate to high alcohol consumption was negatively associated with developing type 2 diabetes in the participants with BMI ≥ 22 kg/m^2^, but not in the participants with BMI < 22 kg/m^2^. Watanabe et al.^47^ reported that alcohol consumption was positively associated with developing type 2 diabetes in individuals with BMI < 22 kg/m^2^, but not in those with BMI ≥ 25 kg/m^2^. In summary, alcohol consumption might have positive association with developing type 2 diabetes in individuals with a BMI ≤ 22 kg/m^2^, but may not have an impact on those with ≥ 25 kg/m^2^. However, previous reports had serious limitations, including small sample size, studies with short observation periods, no consideration of blood test data, and failure to consider the observation period in the analysis.

The present study included only Japanese people and showed results different from the association between alcohol consumption and the onset of type 2 diabetes in Western populations. There were several reasons for this observation. Even among obese individuals, the BMI of Japanese people tends to be lower than that of Western people. Therefore, the impact of alcohol consumption may differ between Japanese and Western populations. Ethanol, which is the main component of alcoholic beverages, decomposes into acetaldehyde. Aldehyde dehydrogenase 2 (ALDH2) is required for acetaldehyde decomposition. The ALDH2 allele appears to be most prevalent in Japanese, Chinese-American, Taiwanese, Han Chinese, Koreans, and many Japanese people who are unable to metabolize alcohol adequately compared to other racial groups, such as Western people^48^. Differences in ethanol metabolism might be involved in the variations in the effects of alcohol consumption on the onset of diabetes. Moreover, a unique Japanese food culture has evolved, comprising Japanese cuisine and sake. In particular, ethyl-α-D-glucoside, which is present in sake, has been found to prevent product of interleukin 6 and liver injury^49^. A previous study showed that the intake of sake lees extract improved insulin resistance via the improvement of hepatic inflammation in an animal model^50^. Therefore, alcohol consumption, including sake, might have negative association with the development of type 2 diabetes in the Japanese participants with BMI ≥ 25 kg/m^2^.

Previous report suggested that hypertension and dyslipidemia are implicated in the onset of type 2 diabetes^51^. However, in the current study, hypertension and dyslipidemia were not included as covariates. This decision was made because we believe that alcohol consumption may also impact blood pressure and lipid levels, suggesting that they could serve as intermediate factors in the onset of type 2 diabetes in our analyses.

The present study has certain limitations. First, it was an observational study. Consequently, not only unknown confounding factors but also measurement errors, especially self-reported alcohol consumption, may have existed. Second, the data on dietary intake were not considered. A previous study reported that significantly more food was consumed in the alcohol condition than in the no-alcohol condition^52^. Moreover, socioeconomic factors such as education and income levels, which could affect the development of type 2 diabetes, were not considered. Third, we were unable to sufficiently investigate the association with developing type 2 diabetes associated with alcohol consumption exceeding 66 g/day because any amount greater than 66 g/day is included in the category of ≥ 66 g/day based on the questionnaire format. Fourth, some types of alcohol contain additional bioactive components which may have affected the outcome. However, the type of alcohol the participants consumed was not recorded in this study. Additionally, we evaluated weight of alcohol consumed as a measure in this study. Other measures such as weight of alcohol as a ratio with weight of participant may have affected the outcome. Moreover, drinking habits are more prone to change over time. However, we used only baseline measurement of alcohol consumption. Fifth, previous report suggested that ALDH2 was associated with glucose metabolism in non-obese, non-diabetic Japanese^53^. Unfortunately, however, we have no data about the ALDH2. Sixth, we did not consider the competing risks, such as the incidence of other critical diseases, including cardiovascular and inflammatory diseases, in this study. Seventh, in this study, we evaluated the association between alcohol consumption levels and incidence of type 2 diabetes according to BMI category. However, BMI may not be the most accurate measure of adiposity, particularly in Asian populations. Finally, this study included only middle-aged Japanese employees. Therefore, it is unclear whether our results are applicable to other ethnic groups or populations.

This large-scale and long-term study identified that light alcohol consumption might have negative association with developing type 2 diabetes in middle-aged Japanese. Moderate-to-high alcohol consumption might have positive association with developing type 2 diabetes in Japanese with a BMI < 25 kg/m^2^. Alcohol consumption might have negative association with the development of type 2 diabetes in Japanese with BMI ≥ 25 kg/m^2^. However, there is evidence that alcohol consumption in any quantity has harmful effects^54^. We emphasize that the results of this study do not recommend alcohol consumption for the prevention of diabetes, and when consuming alcohol, responsible intake is recommended because alcohol consumption may induce alcoholic steatohepatitis, pancreatitis, cancers, dementia, cerebral hemorrhage, and mental disorders. Large prospective trials and more observational studies are needed to better assess the relationship between alcohol consumption and the development of type 2 diabetes in Japanese.

## Methods

### Study design

This long-term retrospective cohort study used data from 2008 to 2021 obtained from the Panasonic cohort study database, which included data on annual medical health checkups, medical costs, medical history, and mortality conducted by Panasonic Corporation, Osaka, Japan. All workers underwent annual medical health check-ups. The goal of this program is to improve public health by early detection of chronic illnesses, including metabolic disorders, and assessment of underlying risk factors.

Following a more than 10-h fast, blood samples were taken. The baseline attributes were evaluated using a standardized and validated self-administered questionnaire. Participants were categorized as nonsmokers, past smokers, or current smokers. Individuals were classified as smokers if they smoked at least one cigarette. Regular exercisers were defined as those who engaged in physical activity for at least 30 min, two days a week, for at least a year.

The Panasonic Health Insurance Organization's local ethics committee granted consent for this study (approval number: 2021–001), and it was carried out in conformity with the Declaration of Helsinki's principles. The data was anonymized. Informed consent was obtained using the opt-out method.

### Alcohol consumption

Alcohol consumption was determined using a questionnaire that included the amount consumed per day and frequency per week. The survey assessed the frequency of alcohol consumption using the following questions: "Do not drink at all," "1–3 days a week," "4–6 days a week," and "Every day." Additionally, the daily alcohol intake was assessed with the following questions: "Less than 180 ml of sake," "180–360 ml of sake," "360–540 ml of sake," and "540 ml or more of sake." These formats are a common questionnaire format for alcohol consumption in health checkups in Japan, and the daily ethanol intake was calculated based on the average frequency and amount of alcohol consumed per day, where 180 ml of sake is considered equivalent to 22 g of ethanol. The amount of ethanol was estimated for a person consuming 270 ml of sake in a day and was calculated to be 33 g. “Beer 500 ml” and “wine 180 ml” were converted to the equivalent of “sake 180 ml” by participants using the self-administered questionnaire, thus calculating the ethanol content. Former drinkers were classified as non-drinkers. Alcohol consumption was categorized as 0 g/day, 0 < to < 22 g/day, 22 to < 39 g/day, 39 to < 66 g/day, and ≥ 66 g/day.

### Inclusion and exclusion criteria

Figure 1 displays the study flow diagram for participant registration. Employees aged 40 and above who underwent medical health checkups between 2008 and 2020 were included in this study. We observed the onset of diabetes until 2021. Individuals who did not undergo a blood examination at baseline, those with missing data (BMI, self-administered questionnaires, or alcohol consumption), those with only baseline data, and those with diabetes at baseline were excluded from the study.

### Definition of type 2 diabetes

A fasting plasma glucose (FPG) ≥ 126 mg/dL or being on diabetic therapy was considered as type 2 diabetes^55^. FPG was evaluated at every annual medical health check-up. The diabetic therapy was measured using a self-administered questionnaire. Type 2 diabetes incidence was measured between 2009 and 2021.

### Covariates

The covariates were determined based on a directed acyclic graph from factors previously reported to be associated with the onset of diabetes^51^. Although previous study has reported that factors such as elevated blood pressure and dyslipidemia are also associated with the development of diabetes^51^, in this study, we treated them as intermediate factors.

### Statistical analyses

Potential confounding variables were computed to determine their means and frequencies. The incidence of type 2 diabetes was calculated according to alcohol consumption. Cox regression analysis with multivariable models for interval-censored data was used to assess the relationship between alcohol consumption and the onset of type 2 diabetes. The cox.zph test was used to verify the proportional hazards assumption. Alcohol consumption was divided into five groups based on the baseline self-administered questionnaire: 0 g/day, 0 < to < 22 g/day, 22 to 39 < g/day, 39 to < 66 g/day, and ≥ 66 g/day. HRs were determined using a reference level of 0 g/day of alcohol consumption. The multivariable model was adjusted for BMI, sex, age, smoking status, and exercise habits. Subgroup analyses were performed to assess the effects of baseline BMI, FPG levels, and sex. Because the Japan Society for the Study of Obesity defines obesity as a BMI of ≥ 25 kg/m^2^ and elevated FPG was defined as FPG of ≥ 100 mg/dl^55^, the incidence of type 2 diabetes was stratified according to the categories of BMI < 25 kg/m^2^ and BMI ≥ 25 kg/m^2^, and FPG < 100 mg/dl and FPG ≥ 100 mg/dl at baseline. Moreover, we also assessed the effects of sex because there are differences in alcohol consumption between sexes. We also tested a potential interaction effect between BMI, FPG categories at baseline and sex, and alcohol consumption by including interaction terms in the Cox proportional hazards model. Considering reverse causation, we concluded sensitivity analysis after excluding incident cases that occurred within the first two years of the follow-up. Moreover, we also conducted a sensitivity analysis, adding BMI as a continuous variable covariate. Additionally, we conducted sensitivity analysis according to BMI category of BMI < 23 kg/m^2^ and ≥ 23 kg/m^2^.

The continuous variables are displayed either as absolute numbers or as mean ± SD. Differences were considered statistically significant at P < 0.05. Associations are presented as HRs with a 95% confidence interval (95% CI). Statistical analyses were performed using the JMP software version 17 (SAS Institute, Cary, NC, USA).

### Supplementary Information

Supplementary Figure 1.

Supplementary Figure 2.

Supplementary Figure 3.

Supplementary Figure 4.

Supplementary Figure 5.

Supplementary Legends.

## Data Availability

If there are legitimate reasons, data will be provided through the corresponding author.
