# Supplementary figures and images for: Association between alcohol consumption and incidence of type 2 diabetes in middle-aged Japanese from Panasonic cohort study 12
Source: Sci Rep. 2024 Sep 2;14:20315. doi: 10.1038/s41598-024-71383-6 (PMC11369267; doi:10.1038/s41598-024-71383-6)

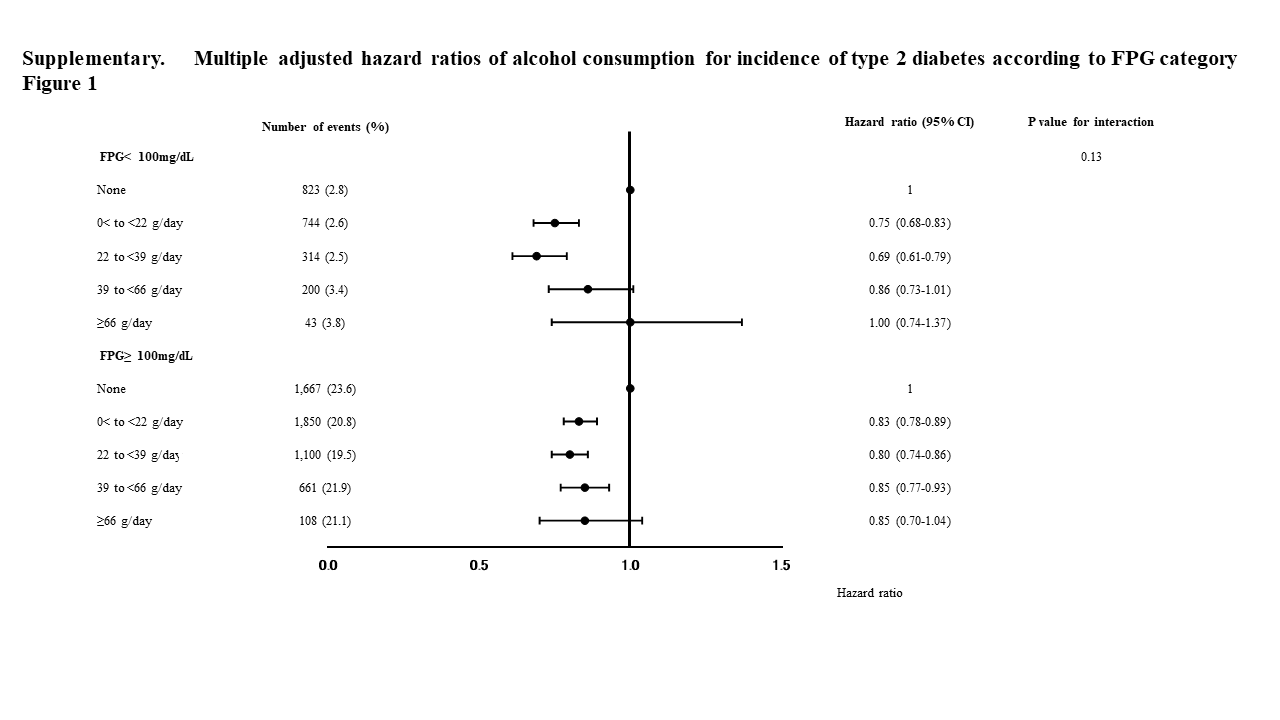

Supplement: Supplementary file 1 — Supplementary Figure 1. [file 41598_2024_71383_MOESM1_ESM.tif]

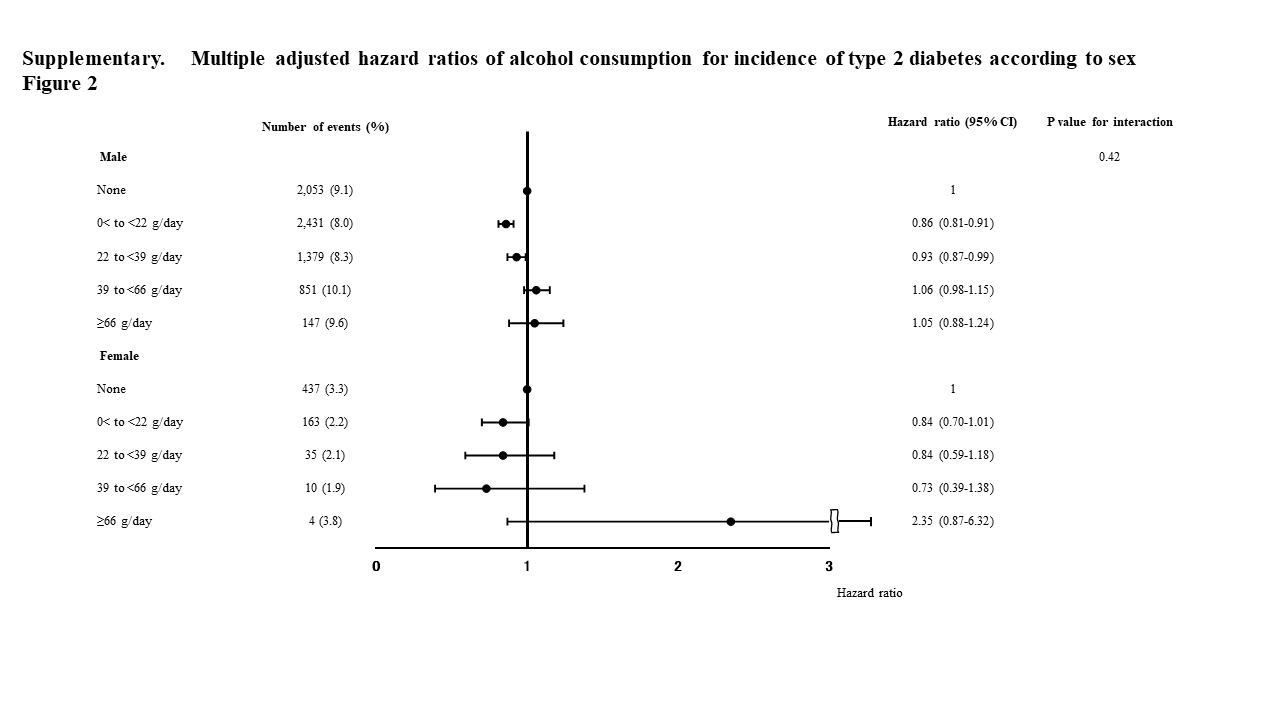

Supplement: Supplementary file 2 — Supplementary Figure 2. [file 41598_2024_71383_MOESM2_ESM.tif]

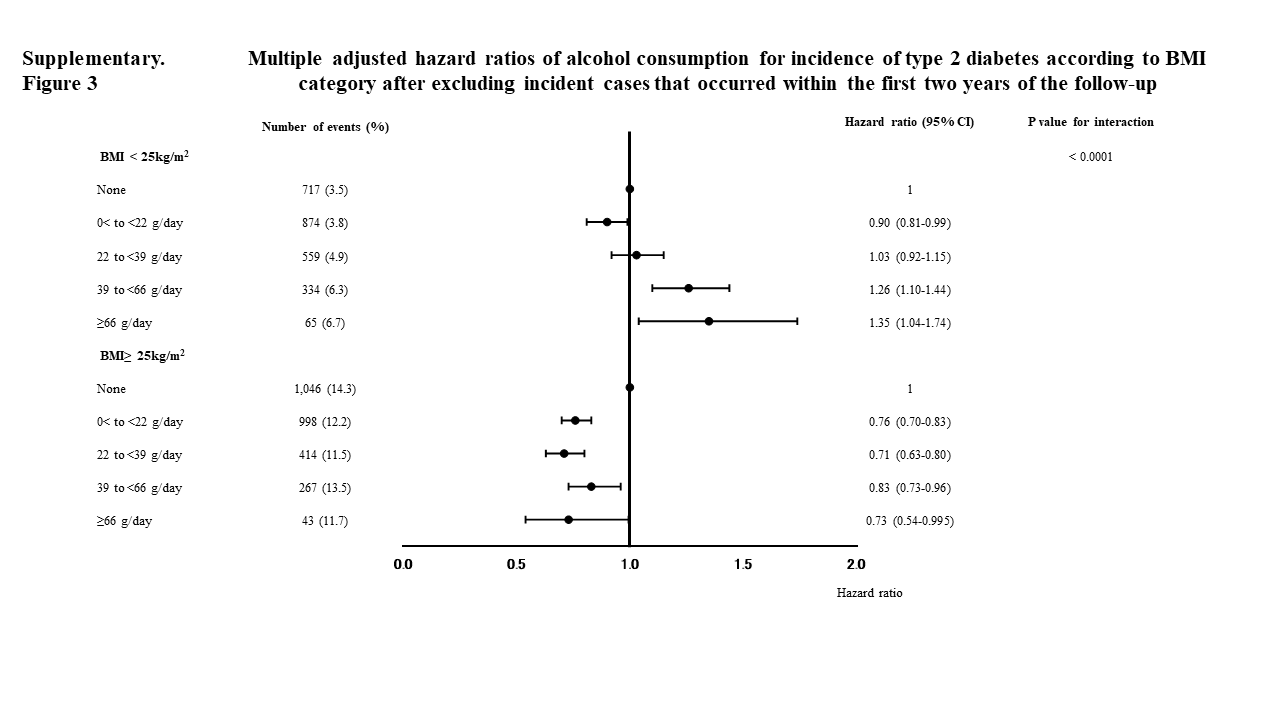

Supplement: Supplementary file 3 — Supplementary Figure 3. [file 41598_2024_71383_MOESM3_ESM.tif]

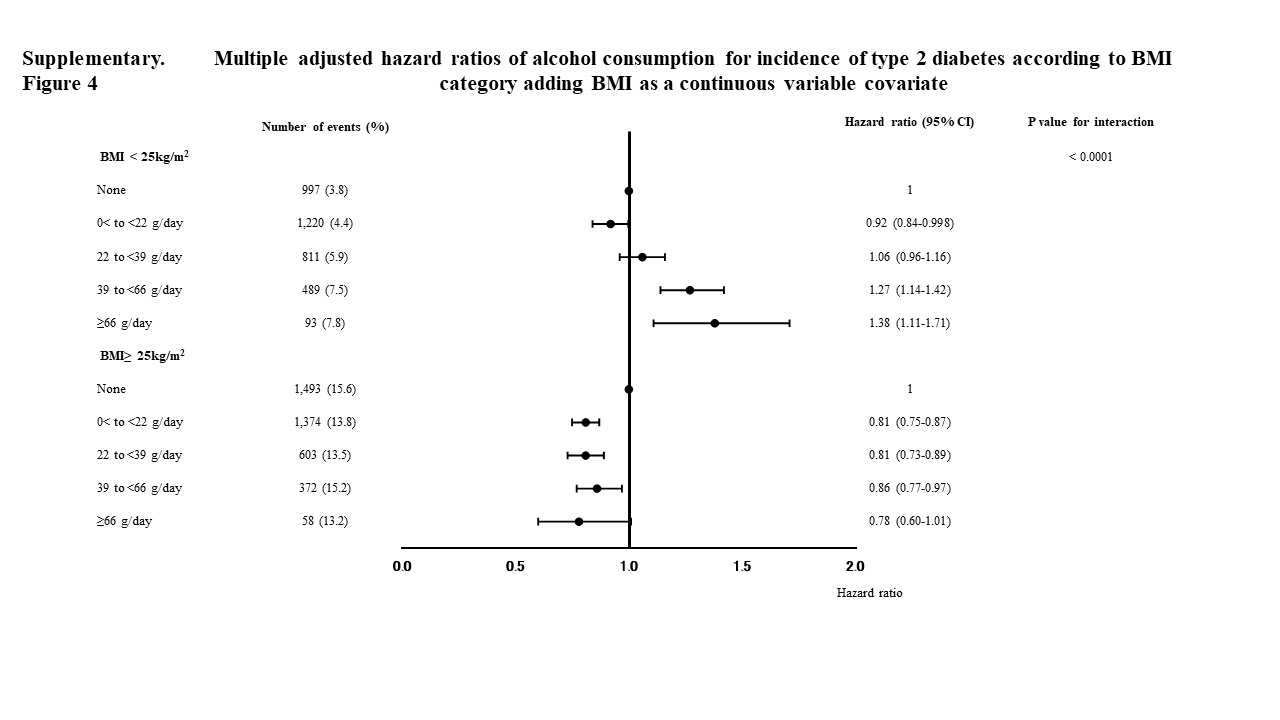

Supplement: Supplementary file 4 — Supplementary Figure 4. [file 41598_2024_71383_MOESM4_ESM.tif]

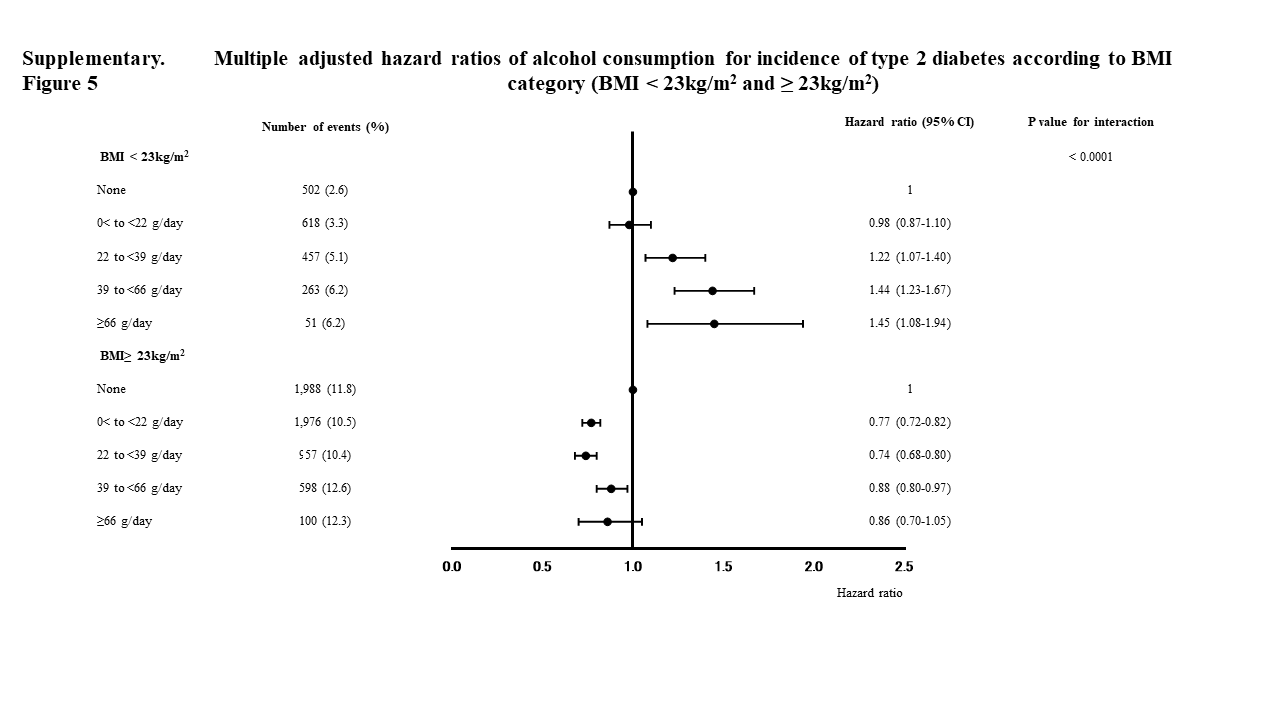

Supplement: Supplementary file 5 — Supplementary Figure 5. [file 41598_2024_71383_MOESM5_ESM.tif]
