# Supplementary material for: Association between alcohol consumption and incidence of type 2 diabetes in middle-aged Japanese from Panasonic cohort study 12
Source: Sci Rep. 2024 Sep 2;14:20315. doi: 10.1038/s41598-024-71383-6 (PMC11369267; doi:10.1038/s41598-024-71383-6)
Supplement: Supplementary file 6 — Supplementary Legends. [file 41598_2024_71383_MOESM6_ESM.docx]

**supplementary Figure 1**

Adjusted hazard ratios of alcohol consumption for incidence of type 2 diabetes based on fasting plasma glucose category adjusted for age, sex, BMI, smoking status, and exercise habits.

**supplementary Figure 2**

Adjusted hazard ratios of alcohol consumption for incidence of type 2 diabetes according to sex adjusted for age, BMI, smoking status, and exercise habits.

**supplementary Figure 3**

Adjusted hazard ratios of alcohol consumption for incidence of type 2 diabetes according to BMI category adjusted for age, sex, smoking status, and exercise habits after excluding incident cases that occurred within the first two years of the follow-up (follow-up duration, 11years)

**supplementary Figure 4**

Adjusted hazard ratios of alcohol consumption for incidence of type 2 diabetes according to BMI category adjusted for age, sex, BMI (continuous variable), smoking status, and exercise habits

**supplementary Figure 5**

Adjusted hazard ratios of alcohol consumption for incidence of type 2 diabetes according to BMI category (BMI < 23kg/m^2^ and ≥ 23kg/m^2^) adjusted for age, sex, smoking status, and exercise habits
